# Identification of key differentially expressed genes and gene mutations in breast ductal carcinoma in situ using RNA-seq analysis

**DOI:** 10.1186/s12957-020-01820-z

**Published:** 2020-03-10

**Authors:** Congyuan Zhu, Hao Hu, Jianping Li, Jingli Wang, Ke Wang, Jingqiu Sun

**Affiliations:** Department of General Surgery, the Affiliated Hospital of Jiangnan University (original area of Wuxi Third People’s Hospital), No. 585 North Xingyuan Road, Liangxi District, Wuxi, 214002 Jiangsu China

**Keywords:** Breast ductal carcinoma in situ, Differentially expressed genes, Mutant genes, RNA-Seq analysis, Transcription factor

## Abstract

**Background:**

The aim of this study was to identify the key differentially expressed genes (DEGs) and high-risk gene mutations in breast ductal carcinoma in situ (DCIS).

**Methods:**

Raw data (GSE36863) were downloaded from the database of Gene Expression Omnibus (GEO), including three DCIS samples (DCIS cell lines MCF10.DCIS, Sum102, and Sum225) and one normal control sample (normal mammary epithelial cell line MCF10A). The DEGs were analyzed using NOIseq and annotated via DAVID. Motif scanning in the promoter region of DEGs was performed via SeqPos. Additionally, single nucleotide variations (SNVs) were identified via GenomeAnalysisTK and SNV risk was assessed via VarioWatch. Mutant genes with a high frequency and risk were validated by RT-PCR analyses.

**Results:**

Finally, 5391, 7073, and 7944 DEGs were identified in DCIS, Sum102, and Sum22 cell lines, respectively, when compared with MCF10A. VENN analysis of the three cell lines revealed 603 upregulated and 1043 downregulated DEGs, including 16 upregulated and 36 downregulated transcription factor (TF) genes. In addition, six TFs each (e.g., *E2F1* and *CREB1*) were found to regulate the core up- and downregulated DEGs, respectively. Furthermore, SNV detection results revealed 1104 (MCF10.DCIS), 2833 (Sum102), and 1132 (Sum22) mutation sites. Four mutant genes (*RWDD4*, *SDHC*, *SEPT7*, and *SFN*) with high frequency and risk were identified. The results of RT-PCR analysis as well as bioinformatics analysis consistently demonstrated that the expression of *RWDD4*, *SDHC*, *SEPT7*, and *SFN* was downregulated in the tumor tissues as compared with that in adjacent non-tumor tissues.

**Conclusions:**

The differentially expressed TFs, TFs regulating DEGs (e.g., *E2F1* and *CREB1*), and high-frequency mutant genes (*RWDD4*, *SDHC*, *SEPT7*, and *SFN*) might play key roles in the pathogenesis of DCIS.

## Background

Breast ductal carcinoma (BDC), a type of breast cancer, is caused by disordered endocrine function in the ovarian tissue [1]. In 2014, there were 252,710 estimated new breast cancer cases and 40,610 deaths caused by breast cancer in the USA [2]. Ductal carcinoma in situ (DCIS) has become a common disease mostly affecting those in their sixth decade of life, accounting for 20–25% of all incident breast malignancies in industrialized countries [3]. DCIS is histologically characterized by the proliferation of malignant epithelial cells around the basement membrane of the mammary ducts [4]. It is becoming increasingly important to shed light on the molecular mechanisms underlying DCIS.

Remarkable developments in genomic technologies have made researchers decipher the genetic changes that occur in cancer. For example, *RBBP7* (RbAp46) and *BIRC5* (survivin) expression in carcinoma cells is significantly higher in estrogen receptor (ER)-positive pure DCIS than in invasive ductal carcinoma (IDC) [5]. Genes involved in cell signaling and adhesion (*TMEM45A*, *FAT1*, and *DST*) in DCIS have been found to be closely correlated with the transition from in situ cancer to invasive cancer [6]. Furthermore, gene mutations in DCIS have also been associated with the progression of DCIS; these include *BRCA1/2* deleterious mutation [7], somatic mutations in AKT1, *PIK3CA*, and *TP53* [8], and hypermethylation of *HOXA5* and *SOX* genes [9].

In recent years, next-generation sequencing-based approaches, such as RNA-Sequencing (RNA-Seq), have garnered the potential to offer unprecedented in-depth analysis of gene expression. Based on RNA-Seq analysis, Tian et al. [10] identified differentially expressed transcripts in DCIS models, which are associated with signaling pathways such as cell proliferation, cell-cell adhesion, and cell-cell signaling, and reported that aldehyde dehydrogenase 5A1 (ALDH5A1) may serve as a novel molecular target in DCIS treatment. However, genetic mutations associated with DCIS were not investigated. In the present study, RNA-Seq data deposited by Kaur et al. [11], including three DCIS samples (MCF10.DCIS, Sum102, and Sum22) and one normal control sample (MCF10A), were downloaded to identify the differentially expressed genes (DEGs). After functional annotation of the DEGs, motif finding was performed in the promoter region of DEGs. Additionally, single nucleotide variants (SNVs) in DCIS were identified and annotated. These results might improve our understanding of the molecular mechanism underlying DCIS and suggest potential targets for clinical treatment.

## Materials and methods

### Data sources of RNA-seq

The RNA-seq data GSE36863 [11] were downloaded from the database of Gene Expression Omnibus (GEO), including three DCIS samples (DCIS cell lines MCF10.DCIS (GSM903304), Sum102 (GSM903305), and Sum22 (GSM903306)), and one normal control sample (mammary epithelial cell MCF10A (GSM903303)). The samples were subjected to 76 cycles of single-end sequencing via an Illumina Genome Analyzer GAIIx (Illumina Inc., San Diego, California, USA). Two biological replicates of the three DCIS models (Sum102, MCF10.DCIS, and Sum225) and the MCF10A model were used to perform whole transcriptome sequencing.

MCF10.DCIS and MCF10A cell lines were obtained from the Cell Lines Resource (Karmanos Cancer Institute, Detroit, MI). Sum225 and Sum102 cell lines were provided by Dr. Stephen Ethier (Hollings Cancer Center, Charleston, SC). Sum225 cells were isolated from a chest wall recurrence in a DCIS patient. The Sum102 cell line was isolated from a patient who was diagnosed with extensive DCIS with areas of micro-invasion. The four cell lines were grown in three dimensional (3D) reconstituted basement membrane (rBM) overlay culture for 12 days and the culture medium was changed every 4 days.

### Read alignment and differential expression analysis

All the RNA-seq data were mapped to the reference human genome (hg19) in University of California Santa Cruz database using TopHat [12]. For each read, mismatches ≤ two bases were permitted, and other parameters followed the default settings. After read alignment, on the basis of the gene annotation data in the reference sequence (Refseq) database, the transcripts were assembled using Cufflinks [13], and the gene expression levels of the transcripts were calculated using fragments/kilobase/million reads method in Cuffdiff [13]. DEGs between DCIS and normal samples were then selected via NOISeq [14] with a *q* value ≥ 0.8.

### Functional annotation of DEGs

The Database for Annotation, Visualization and Integrated Discovery (DAVID) [15] was used to analyze the Gene Ontology (GO) functions, including biological process (BP), molecular function (MF), and cellular component (CC), of DEGs. A *p* value < 0.05 was set as the criterion. Then, DEGs with transcription factor (TF) functions were screened and annotated based on the TRANSFAC database (http://www.genomatix.de). Ultimately, according to the tumor suppressor genes (TSGs) in the TSgene database [16] and the tumor associated genes (TAGs) [17], known TSGs, and oncogenes were identified in the DEGs.

### Examination of upstream regulatory element of DEGs

The VENN test was conducted to select the co-expressed DEGs in the MCF10.DCIS, Sum102, and Sum225 cell lines. In the promoter region (from 1 kb upstream to 0.5 kb downstream of transcription initial site) of the up- and downregulated DEGs, motif finding was performed using Seqpos [18] to predict their potential TFs. A *p* value < 0.00001 and frequency > 50% were set as cutoff criteria.

### SNV detection

SNVs were detected using GenomeAnalysisTK [19] with the criteria of coverage > 5 and quality score ≥ 30. Especially, SNVs with a quality score of > 50 were defined as the high-reliability SNVs. On the basis of the known single nucleotide polymorphisms (SNPs) recorded in the dbSNP137 database and 1000 Genomes Project, the known SNPs in the SNVs were removed. Besides, SNV calling was optimized by using the RNA-seq data of the normal sample, and accordingly, RNA editing in the transcriptome was removed effectively.

### Annotation of somatic mutant sites and detection of high-risk mutation

VarioWatch can provide the annotations of two human genome reference versions (NCBI build 36.3, NCBI build 37.2), including gene annotations, known variants from dbSNP, pre-computed variation risks, 1000 Genomes Project (released on October 2011), OMIM (T), and other minor variant databases [20]. In this study, VarioWatch was applied to annotate the SNVs in the coding regions of the mutant genes and analyze their corresponding influence on the protein products. The genes with high-risk base mutations in each sample, and their frequency of occurrence in the three cell lines, were also analyzed. The mutations that occurred in more than 2 cell lines were defined as high-frequency mutations.

### Real-time polymerase chain reaction verification

RT-PCR was used to verify and compare the expression levels of four mutated genes (*RWDD4*, *SDHC*, *SEPT7*, and *SFN*) between tumor tissues and adjacent non-tumor tissues. Total cellular RNA was extracted with 1 ml TRIzol reagent (9109, TaKaRa), and RNA purity was detected using a microplate reader (Infinite M100 PRO, TECAN). One microgram of RNA was reverse-transcribed into cDNA using 4 μl 5× PrimeScript RT Master MIX (RR036A, TAKARA). RT-PCR was conducted on the ABI Prism 7900HT Fast Real-Time PCR system using the specific primer pairs as described in Table 1 and SYBR Premix EX Taq (A25742, Thermo). The protocol was set as follows: 50 °C for 2 min, 95 °C for 10 min, 40 cycles of 95 °C for 10 s, 60 °C for 30 s. All the samples were normalized to the corresponding expression of internal control *GAPDH*. The test was performed in triplicate and the relative expression levels were calculated with the 2^-ΔΔ^Ct method. *p* < 0.05 and *p* < 0.01 respectively indicated a significant difference and extremely significant difference.

**Table 1:** Primers and primer sequences of Q-PCR genes

Primer	Sequence(5′–3′)
*RWDD4*-hF	TGCCAACGAGGACCAGGAG
*RWDD4*-hR	AGGCTTTGGGATCACCATTTT
*SDHC*-hF	TGCCTCCGAGCCCACTTTA
*SDHC*-hR	TTATTCCAGAACCGCTCCATC
*SEPT7*-hF	AGGAGCGTCAACAGCAGCAC
*SEPT7*-hR	CCCAATCCAGATTCACCCACT
*SFN*-hF	GCCGAACGCTATGAGGACA
*SFN*-hR	GCTCAATACTGGACAGCACCC
*GAPDH*-hF	TGACAACTTTGGTATCGTGGAAGG
*GAPDH*-hR	AGGCAGGGATGATGTTCTGGAGAG

## Results

### Identification of DEGs and functional enrichment analysis

As compared with the MCF10A cell line, 5391, 7073, and 7944 DEGs were identified in MCF10.DCIS, Sum102, and Sum22 cell lines, respectively, and the ratios of up- and downregulated DEGs were 0.98, 0.99, and 1.26, respectively, without any significant bias of up- or downregulation.

**Fig. 1:**
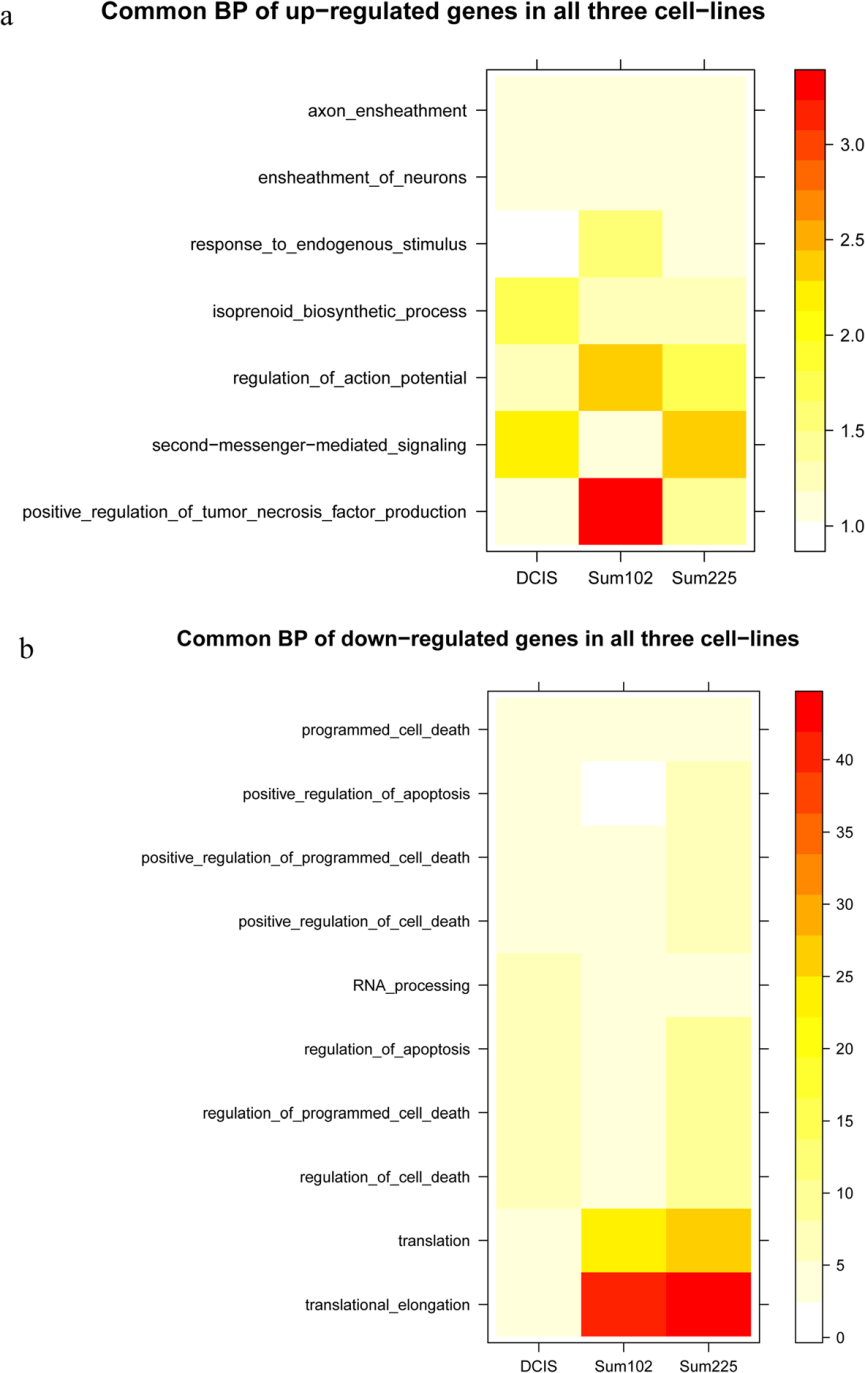
Functional enrichment analysis of up- and downregulated genes in MCF10.DCIS, Sum102, and Sum225 cell lines. **a** Functional enrichment analysis of upregulated genes. **b** Functional enrichment analysis of downregulated genes. Colors in every cell represent the significance of functional enrichment in the cell lines. Significance is proportionate to color depth

Subsequently, a functional enrichment analysis of up- and downregulated DEGs in each DCIS cell line was performed. For the upregulated DEGs, seven abnormal GO terms were enriched, including positive regulation of tumor necrosis factor production, second-messenger-mediated signaling, response to endogenous stimulus, axon and neuron ensheathment, isoprenoid biosynthetic process, and regulation of action potential (Fig. 1a). The significance of these seven GO terms was different among the three cancer cell lines. For example, the enrichment of positively regulated tumor necrosis factor production was most significant in Sum102, while the enrichment of second-messenger-mediated signaling was more common in Sum225 and MCF10.DCIS cell lines.

For the downregulated DEGs, GO terms such as positive regulation of programmed cell death, RNA processing, and response to endogenous stimulus were enriched (Fig. 1b). However, only downregulated DEGs of Sum102 and Sum225 cell lines were enriched in translational elongation, as associated with the regulation of protein translation process.

### Identification of core DEGs and functional enrichment analysis

**Fig. 2:**
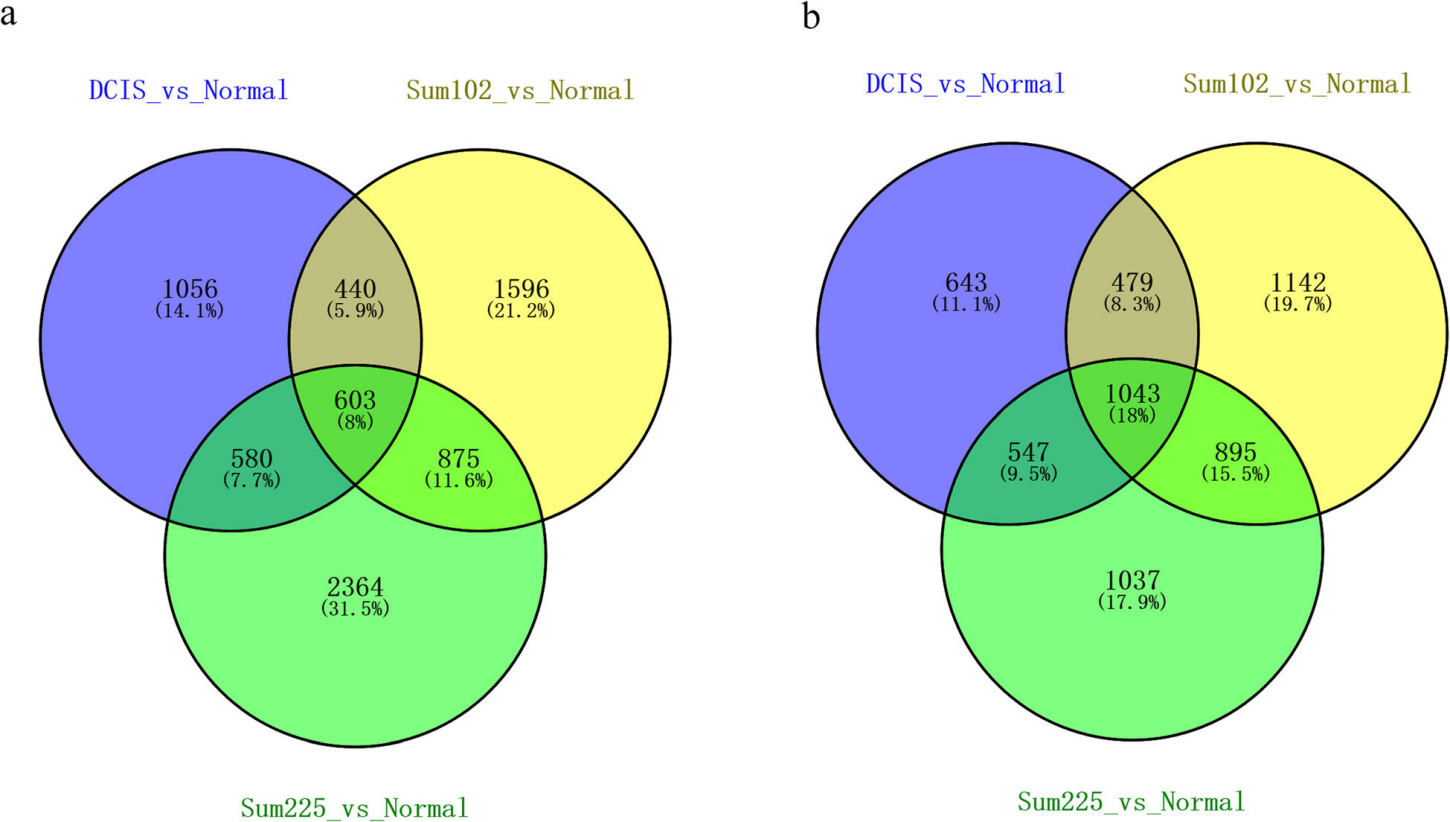
VENN analysis of differentially co-expressed genes in MCF10.DCIS, Sum102, and Sum225 cell lines. **a** Upregulated DEGs in the MCF10.DCIS, Sum102, and Sum225 cell lines. **b** downregulated DEGs in the MCF10.DCIS, Sum102, and Sum225 cell lines.

Through VENN analysis, we detected 603 upregulated and 1043 downregulated DEGs overlapping in the three cancer cell lines (Fig. 2), including 16 upregulated TFs (e.g., *HOXA2*, *HOXB2*, *HOXC10*, and *NKX2-1*) and 36 downregulated TFs (e.g., *BACH1* and *CDX1*), respectively. Herein, these overlapping DEGs were considered as the core DEGs. The upregulated overlapping DEGs were significantly enriched in the regulation of cAMP biosynthetic process, response to lipopolysaccharide, small GTPase-mediated signal transduction, and cytokine production, while the downregulated overlapping DEGs were significantly enriched in the regulation of cell migration, negative regulation of cell proliferation, cell adhesion, and JNK cascade (Table 2).

**Table 2:** Functional enrichment analysis of the overlapping DEGs

GO Term	Gene Counts	TF Genes	*p* value
Downregulated overlapping DEGs			
Translation	38		3.88E-06
Positive regulation of programmed cell death	41	*HIP1*, *ID3*, *IFI16*, *NR3C1*, *PML*, *RXRA*, *TP53BP2*	1.36E-04
RNA processing	46		7.09E-04
Regulation of cell migration	20		8.03E-04
Negative regulation of cell proliferation	33	*ESR2*, *NKX3-1*, *NME2*, *PML*, *RXRA*	0.001239
Regulation of lipid metabolic process	15	*NR3C1*, *NR5A1*, *PPARA*	0.001380
Cell adhesion	52	*LEF1*, *NME2*	0.004558
JNK cascade	9		0.008755
Cellular protein localization	33		0.008962
Response to hormone stimulus	30	*ESR2*, *PPARA*, *RXRA*	0.010205
Upregulated overlapping DEGs			
Positive regulation of tumor necrosis factor production	4		0.005861
Regulation of tumor necrosis factor production	5		0.007442
Response to lipopolysaccharide	7	*NKX2-1*	0.013341
Small GTPase mediated signal transduction	15		0.024022
Cytokine production	5		0.028739
Regulation of phosphate metabolic process	20		0.040473
Regulation of cAMP biosynthetic process	7		0.043362
Blood circulation	10		0.047845

*DEGs* differentially expressed genes, *TF* transcription factor, *GO* gene ontology

According to the analysis of cellular localization, 11 core upregulated DEGs (e.g., *FREM2* and NOD2) and 17 core downregulated DEGs (e.g., *PTPRK* and *CAV1*) were associated with the cell surface, suggesting that the characteristics of cell surface proteins of the DCIS cells could help distinguish DCIS cells from normal cells. Additionally, the protein products of 5 upregulated DEGs (*LAMB4*, *COL9A2*, *FREM2*, and *COL10A1*) and 12 downregulated DEGs (e.g., *COL17A1*, *COL7A1*, and *LAMA5*) were specifically located in the ECM, indicating that DCIS might be diagnosed via the detection of specific protein factors in blood (Table 3).

**Table 3:** The overlapping DEGs associated with cell surface and extracellular matrix part

Location	Upregulated DEGs		Downregulated DEGs	
	Count	Gene name	Counts	Gene name
Cell surface	11	NOD2, IL12RB1, CORIN, *FREM2*, FLT3, LRRTM1, FCER1G, CHRNA4, TPO, HSPA5, SCNN1A	17	*PTPRK*, *CAV1*, *PARD3*, *SELL*, *LHCGR*, *MFGE8*, *ITGA4*, *ADA*, *CDH13*, *SDC1*, *BGN*, *SULF2*, *RC3H2*, *HSPB1*, *TGFBR3*, *SCNN1G*, *ADAM9*
Extracellular matrix part	5	LAMB4, COL9A2, *FREM2*, COL11A2, COL10A1	12	*COL17A1*, *COL7A1*, *LAMA5*, *NID1*, *COL1A1*, *LAMB1*, *DST*, *PRSS12*, *COL4A6*, *COL4A5*, *FN1*, *ANXA2*

*DEGs* differentially expressed genes

### Upstream TFs and functional enrichment analysis of DEGs

According to motif scanning, six TF motifs each were enriched in the promoter regions of the core up- and downregulated DEGs (Table 4). Especially, *CREB1* and *E2F1* motifs were identified in the promoters of the upregulated overlapping DEGs.

**Table 4:** Upstream TF analysis of overlapping DEGs

Promoter regions	Candidate TF counts	TF genes
Downregulated DEGs	6	*E2F1*, *ELK1*, *HIF1A*, *MYB*, *NR2C2*, *TFAP2A*
Upregulated DEGs	6	*CREB1*, *ELK4*, *GABPA*, *NFYA*, *PAX5*, *ZBTB7B*

*DEGs* differentially expressed genes *TF* transcription factor

### Detection of somatic mutations and SNVs

**Fig. 3:**
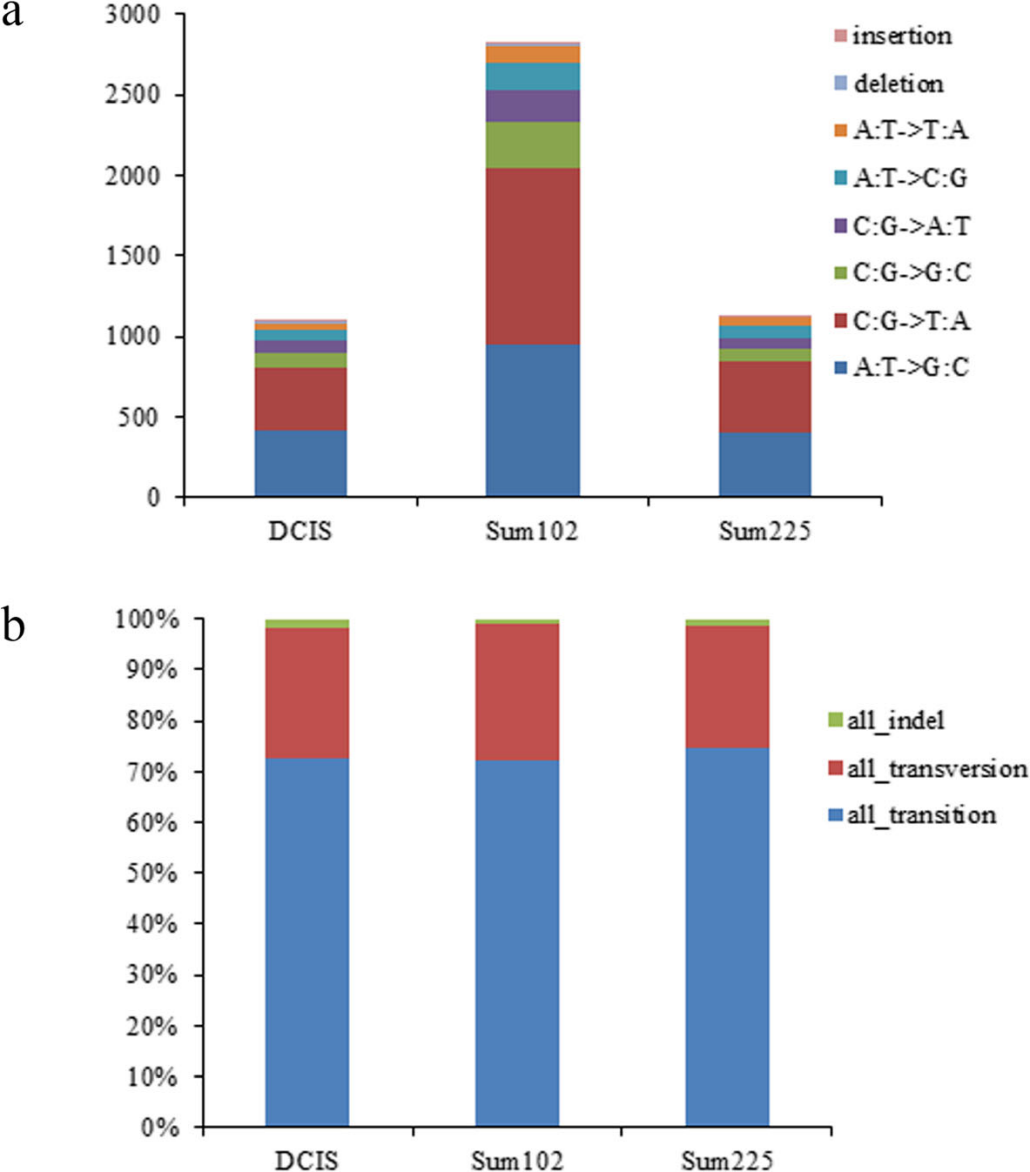
Statistics of single nucleotide variations in MCF10.DCIS, Sum102, and Sum225 cell lines. **a** Total number of various mutations in samples. **b** Percentages of various mutations in samples

In total, 1104, 2833, and 1132 somatic mutations were identified in MCF10.DCIS, Sum102, and Sum225 cell lines, respectively (Fig. 3a). For each cell line, transition was the main mutation type (> 72%), the frequency of transversion was approximately 25%, and the frequency of insertion-deletion (indel) was only 3 % (Fig. 3b). A total of 19, 31, and 17 indel sites were detected in MCF10.DCIS, Sum102, and Sum225 cell lines, respectively. Furthermore, four high-frequency mutant genes were identified according to the criteria in at least two cell lines (Table 5). Base mutations located in chr1:161332189 and chr1:27190350 led to missense mutations in *SDHC* and *SFN*, respectively. Base mutations in chr4:184562622 and chr7:35840881 were located at the exon splicing sites and led to an abnormal post-transcriptional splicing of *RWDD4* and *SEPT7*. *SDHC* mutations occurred in Sum102 and Sum225 cell lines at the same mutant site, as well as *SFN*. *RWDD4* and *SEPT7* mutations occurred in both MCF10.DCIS and Sum102 cell lines.

**Table 5:** Mutant sites with high-frequency in breast ductal carcinoma in situ cell lines

Chromosome	Position	Reference base	Mutated base	Corresponding gene	Observed frequency	Tumor-associated genes
chr1	161332189	C	A	*SDHC*	2	
chr1	27190350	G	A	*SFN*	2	Tumor suppressor
chr4	184562622	C	A	*RWDD4*	2	
chr7	35840881	G	C	*SEPT7*	2	

### RT-PCR verification

**Fig. 4:**
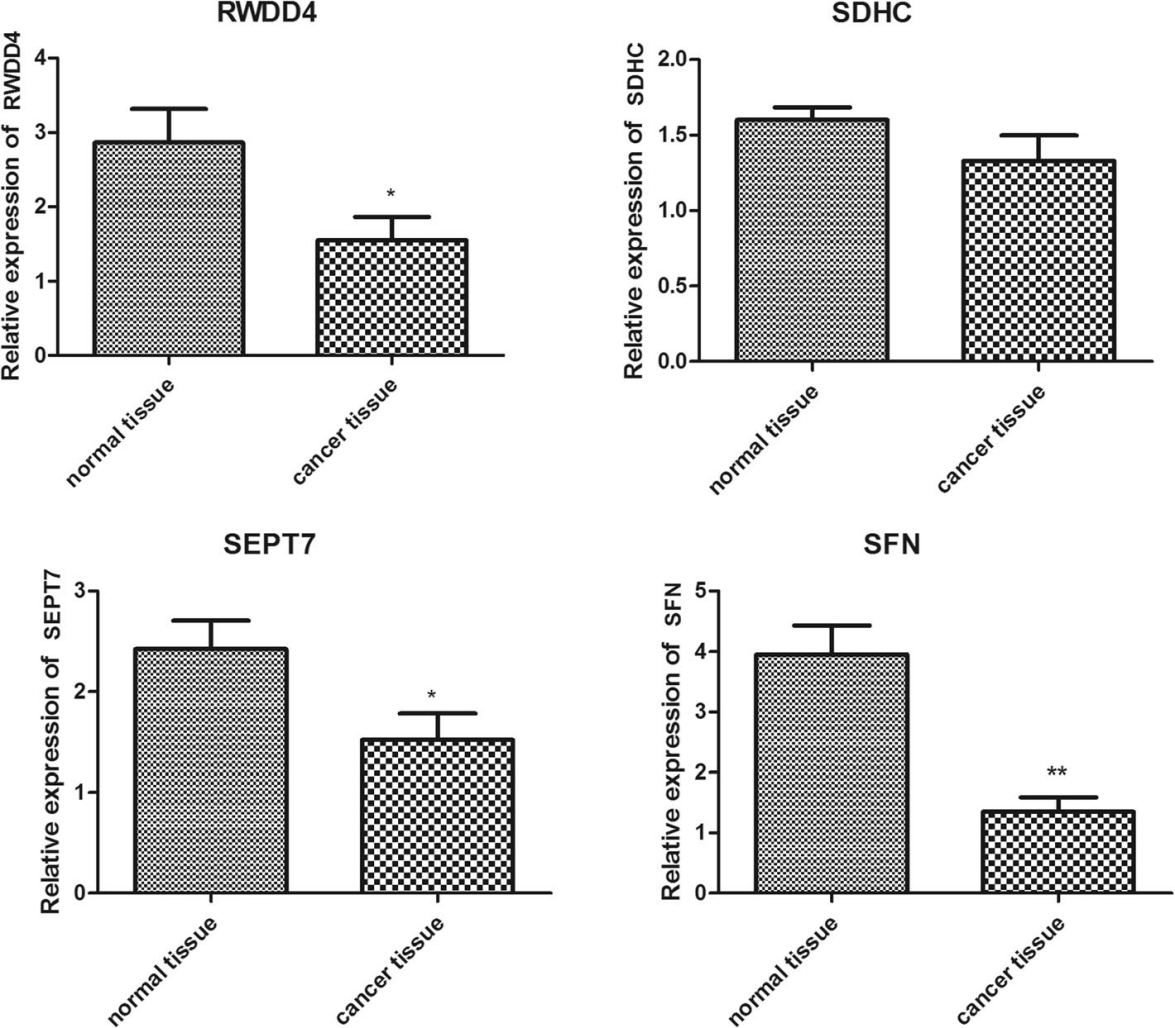
Verification results of real-time polymerase chain reaction (RT-PCR) of *RWDD4*, *SDHC*, *SEPT7*, and *SFN*. * Indicates a significant difference between normal tissue and cancer tissue (*p* < 0.05). ** Indicates an extremely significant difference between normal tissue and cancer tissue (*p* < 0.01)

Consistent with the bioinformatics analysis results, the RT-PCR results revealed an overall downregulation. As shown in Fig. 4, the expression levels of *RWDD4*, *SEPT7*, and *SFN* were significantly lower in the breast cancer tissues than in the adjacent non-tumor tissues (*p* < 0.05 for *RWDD4*, *p* < 0.05 for *SEPT7*, *p* < 0.01 for *SFN*). Although the expression level of *SDHC* was not significantly downregulated in the breast cancer tissues, the trend of *SDHC* expression was consistent with that of *RWDD4*, *SEPT7*, and *SFN* expression.

## Discussion

In this study, based on the RNA-seq data of DCIS, 603 core upregulated and 1043 core downregulated DEGs were identified among the three DCIS cell lines. Among them, there were 16 upregulated and 36 downregulated TFs. Furthermore, the products of 11 upregulated and 17 downregulated DEGs were located on the cell surface, and the products of 5 upregulated and 12 downregulated DEGs were located in the ECM. In addition, six TF motifs each were enriched in the promoter regions of core up- and downregulated DEGs, respectively. Additionally, four high-frequency mutant genes were detected.

Among the core DEGs, the upregulated genes, such as *HOXA2*, *HOXB2*, *HOXC10*, and *NKX2-1*, were identified as TFs. *HOXA2*, *HOXB2*, and *HOXC10* belong to the homeobox family of genes, which play an important role in morphogenesis and differentiation [21]. A previous study had reported that some homeobox genes, such as *HOXB13*, *TLX1*, and *HNF1B*, were hypermethylated in the early stages of breast cancer [22]. *HOXB2* and *HOXC10* were identified to be differentially expressed in the hyperplastic enlarged lobular unit when compared with the normal terminal duct lobular unit, and other homeobox genes (e.g., *HOXA4* and *HOXB6*) were differentially expressed in DCISs with respect to normal tissues [23]. In addition, DCIS is closely associated with the silencing of *HOXA2* [23]. As a result, the homeobox genes *HOXA2*, *HOXB2*, and *HOXC10* may be closely correlated with DCIS. *NKX2-1* (NK2 homeobox 1, also known as TITF1/TTF-1) is a thyroid-specific transcription factor, which regulates the expressions of thyroid-specific genes as well as genes involved in morphogenesis [24]. There is evidence that TTF-1 stain is negative in the DCIS cells [25], suggesting that *NKX2-1* may play a crucial role.

Furthermore, the protein encoded by *FREM2* (FRAS1 related extracellular matrix protein 2) was detected to be upregulated on the cell surface and in the ECM of DCIS tissue. *FREM2*, an integral membrane protein, plays a role in epidermal-dermal interactions [26]. *FREM2* has been identified as a target gene of TFAP2C (transcription factor AP-2 gamma) in hormone-responsive breast cancer cells [27]. However, no study has reported the association of *FREM2* with DCIS so far. Thus, we speculated that *FREM2* may play a pivotal role in DCIS, by participating in cell interactions.

Based on the motif scanning, *E2F1* was identified as an upstream TF for the core downregulated DEGs. *E2F1* (E2F transcription factor 1) is a target of c-Myc that promotes the cell cycle [28]. It has been demonstrated that E2F-1 expression is significantly higher in DCIS than in the normal breast tissue [29]. Moreover, *E2F1* is involved in ARF tumor suppressor expression and activation in DCIS [30, 31]. Collectively, the dysfunction of the transcriptional regulatory pathway of *E2F1* may contribute to cell multiplication in DCIS. Additionally, the *CREB1* (cAMP response element-binding protein 1) motif was identified in the promoter regions of the upregulated overlapping DEGs. *CREB1* belongs to a subfamily of the leucine zipper with basic domain family of cellular transcription factors. This gene has been detected to be expressed at a high level in breast cancer cell lines MCF7 and MDA-MB-231 and is linked to patients’ overall and disease-free survival [32]. Therefore, *CREB1* may participate in the progression of DCIS via regulation of downstream genes.

In the present study, four high-frequency mutant genes (*RWDD4*, *SDHC*, *SEPT7* and *SFN*) were identified. *RWDD4* (RWD domain-containing protein 4) is a member of the RWD domain protein superfamily [33]. RWDD4 contains a RWD domain that is involved in protein-protein interactions [34]. The knockdown of RWDD4 inhibits transitional cell carcinoma (TCC), cell proliferation, migration, and invasion [35]. However, no study has revealed *RWDD4* mutations in DCIS so far. *SDHC* encodes one of the four nuclear-encoded subunits that comprise succinate dehydrogenase, a mitochondrial protein. *SDHC* mutation has been confirmed to be associated with various cancers, such as kidney cancer [36], gastrointestinal stromal tumors [37], pheochromocytoma [38], and head and neck paraganglioma [39]. There is no evidence that *SDHC* mutation is linked to DCIS, although studies have shown the association of *SDHC* homologs with breast cancer. *SDHB* and *SDHD* variants are highly related to the increased prevalence of breast cancers [40]. Furthermore, a previous study has reported loss of *SDHA* or *SDHB* expression in 3% of breast cancers [41]. The study also suggested that the downregulation of SDHC promoted epithelial to mesenchymal transition, which was accompanied by the structural remodeling of mitochondrial organelles. This may confer a survival benefit upon exposure to a harmful microenvironment during cancer progression [42]. Therefore, we speculated that the *SDHC* mutation might be closely related to the progression of DCIS. *SEPT7* encodes septin 7, which is highly similar to the *CDC10* protein of *Saccharomyces cerevisiae* [43]. There has been no evidence to support the relationship between *SEPT7* and DCIS so far, while DNA methylation-induced altered expression of *SEPT9* has been observed during breast tumorigenesis [44]. The study revealed that SEPT2 and SEPT7 play an important role in cell migration and invasion in breast cancers by controlling the activation of MEK/ERK MAPKs, and that targeting septin proteins may provide a new direction for breast cancer treatment [45]. Thus, *SEPT7* might be involved in DCIS. *SFN* (stratifin, also known as 14-3-sigma) has been found to be hypermethylated and related to cell-cycle regulation in human breast cancer cells [46]. The previous study by Li et al. demonstrated that isothiocyanate SFN treatment inhibited DCIS stem-like cells in vivo and in vitro [47]. Li et al. reported that *SFN* treatment reprogrammed DCIS stem-like cells, as evidenced by the significant changes in exosomal secretion, which is more closely resembling that of the non-stem cancer cells [48]. Therefore, *SFN* might be involved in DCIS.

## Conclusions

In conclusion, differentially expressed TFs (e.g., *HOXA2*, *HOXB2*, *HOXC10*, and *NKX2-1*), genes that encode proteins located on the cell surface and in the ECM of DCIS (e.g., *FREM2*), and TFs of DEGs (e.g., *E2F1* and *CREB1*), as well as high-frequency mutant genes (*RWDD4*, *SDHC*, *SEPT7*, and *SFN*), may participate in the progression of DCIS. These findings may contribute to a better understanding of the molecular mechanism underlying DCIS.

## Data Availability

The datasets used and/or analyzed during the current study are available from the corresponding author on reasonable request.

## Abbreviations

ALDH5A1: Aldehyde dehydrogenase 5A1
BDC: Breast ductal carcinoma
BP: Biological process
CC: Cellular component
DAVID: The Database for Annotation, Visualization and Integrated Discovery
DCIS: Ductal carcinoma in situ
DEGs: Differentially expressed genes
GEO: Gene Expression Omnibus
IDC: Invasive ductal carcinomas
MF: Molecular function
NCBI: National Center for Biotechnology Information
SNPs: Single nucleotide polymorphisms
SNVs: Single nucleotide variations
TAGs: Tumor-associated genes
TF: Transcription factor
TSGs: Tumor suppressor genes
